# Peptide Antibiotic–Polyphosphate Nanoparticles: A Promising Strategy to Overcome the Enzymatic and Mucus Barrier of the Intestine

**DOI:** 10.1021/acs.biomac.3c00083

**Published:** 2023-05-24

**Authors:** Ahmad Saleh, Zeynep Burcu Akkuş-Dağdeviren, Soheil Haddadzadegan, Richard Wibel, Andreas Bernkop-Schnürch

**Affiliations:** † Center for Chemistry and Biomedicine, Department of Pharmaceutical Technology, Institute of Pharmacy, University of Innsbruck, Innrain 80/82, 6020 Innsbruck, Austria; ‡ Department of Pharmacy, Universitas Mandala Waluya, A.H.Nasution, Kendari 93231, Southeast Sulawesi Republic of Indonesia

## Abstract

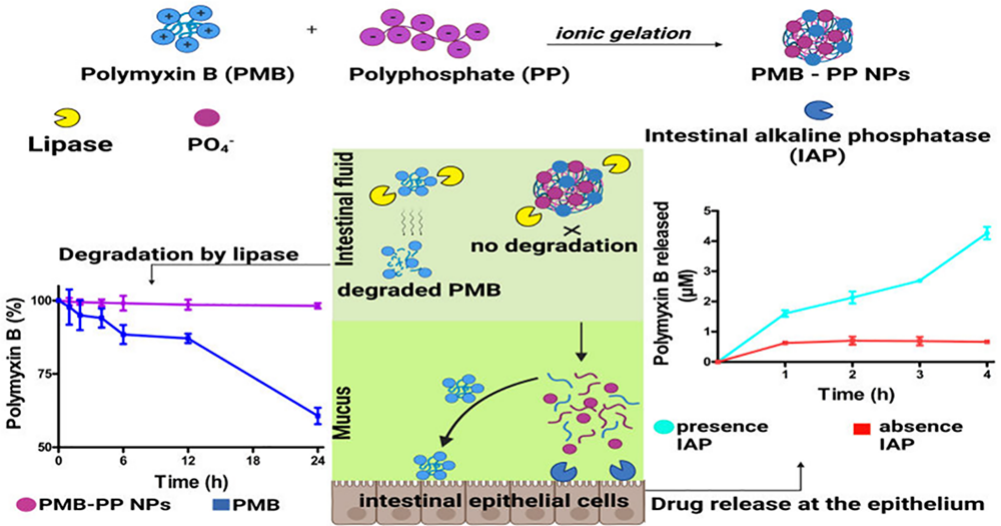

The aim of this study was to develop peptide antibiotic–polyphosphate nanoparticles that are able to overcome the enzymatic and mucus barriers providing a targeted drug release directly on the intestinal epithelium. Polymyxin B–polyphosphate nanoparticles (PMB–PP NPs) were formed via ionic gelation between the cationic peptide and the anionic polyphosphate (PP). The resulting NPs were characterized by particle size, polydispersity index (PDI), zeta potential, and cytotoxicity on Caco-2 cells. The protective effect of these NPs for incorporated PMB was evaluated via enzymatic degradation studies with lipase. Moreover, mucus diffusion of NPs was investigated with porcine intestinal mucus. Isolated intestinal alkaline phosphatase (IAP) was employed to trigger the degradation of NPs and consequent drug release. PMB–PP NPs exhibited an average size of 197.13 ± 14.13 nm, a PDI of 0.36, a zeta potential of −11.1 ± 3.4 mV and a concentration and time-dependent toxicity. They provided entire protection toward enzymatic degradation and exhibited significantly (*p* < 0.05) higher mucus permeating properties than PMB. When incubated with isolated IAP for 4 h, monophosphate and PMB were constantly released from PMB–PP NPs and zeta potential raised up to −1.9 ± 0.61 mV. According to these findings, PMB–PP NPs are promising delivery systems to protect cationic peptide antibiotics against enzymatic degradation, to overcome the mucus barrier and to provide drug release directly at the epithelium.

## 1 Introduction

As the emergence of resistant bacteria endangers the efficacy of most antibiotics, research on new more potent antibiotics and delivery systems for them has been considerably intensified. Among the different classes of antibiotics, polypeptide antibiotics and in particular lipopeptides moved into the limelight of research, as instances of resistance are comparatively rare. With few exceptions, however, these drugs cannot be administrated orally, as they are degraded by gastrointestinal peptidases and lipases and poorly absorbed.^1^ In order to avoid this presystemic metabolism and to release these drugs in a concentrated manner close to the absorption membrane providing a steep concentration gradient as driving force for passive drug uptake, nanoparticles (NPs) are likely the most suitable delivery system.^2,3^ They can protect incorporated drugs toward degradation by gastrointestinal (GI) enzymes and are small enough to permeate the mucus gel layer covering GI epithelia in order to shuttle their payload directly to the absorption membrane. In order to provide a targeted release close to absorption membrane, the membrane-bound enzyme alkaline phosphatase can be utilized as a trigger since it cleaves monophosphates from polyphosphate nanocarriers enabling a release of their cargo at the cellular membrane.^4,5^ So far, however, such NPs have neither been utilized for the oral administration of polypeptide antibiotics nor have polyphosphate nanocarriers been formed without the aid of a polycationic excipient such as chitosan.

The aim of this study was therefore to develop a delivery system protecting polypeptide antibiotics against enzymatic degradation and enabling their transport across the mucus gel layer by the design of polyphosphate NPs. As a model polypeptide antibiotic polymyxin B (PMB) being used for the treatment of multidrug-resistant (MDR) infections in the eye, ear, skin, and bloodstream^6^ was chosen because of its cationic charge and since it can be administrated just via intravenous, intramuscular, pulmonary, intrathecal, and topical routes.^7,8^ Furthermore, various studies have already shown that PMB can be efficiently complexed with anionic polymers forming NPs through electrostatic interactions.^9,10^ The formation of stable complexes between PMB and polyphosphate (PP) should thus be feasible and might enable us to form NPs even without any cationic polymeric excipients that pose a safety concern. Furthermore, a protective effect of the peptide toward enzymatic degradation should be provided. In addition, such NPs might exhibit high mucus permeating properties as the cationic peptide drug cannot be anymore ionically bound to anionic substructures of mucins and their amphoteric surface should even provide mucoinert properties mimicking the surface of highly mucus-diffusive viruses.^11^ Once such NPs have reached the intestinal epithelium, the membrane-bound enzyme intestinal alkaline phosphatase (IAP) is supposed to cleave the polyphosphates to monophosphates triggering the disintegration of NPs and drug release. PMB is a bactericidal drug that acts by disintegrating the outer membrane of Gram-negative bacteria upon destabilization of membrane phospholipids and lipopolysaccharides (LPS). This effect occurs upon electrostatic interactions between cationic PMB and the phosphate groups of the anionic membrane lipids, leading to an increased permeability of the membrane and leakage of intracellular contents resulting in death of bacterial cells.^12^ In order to interact with bacterial cell membranes at the target, PMB should be available in free cationic form. Once PMB–PP NPs are degraded at the target site by IAP, cationic PMB is released interacting with the anionic membrane lipids of bacteria.

Within this study, polymyxin B–polyphosphate NPs (PMB–PP NPs) are developed and characterized by particle size, PDI, and zeta potential. Furthermore, cytotoxicity of NPs is determined on Caco-2 cells via resazurin assay. The protective effect of these NPs toward drug degradation by lipase and their ability to permeate the mucus gel layer is evaluated. Moreover, a targeted drug release directly on the surface of epithelial cells is monitored by incubation of NPs with the cellular membrane-bound enzyme IAP.

## 2 Materials and Methods

### 2.1 Materials

Polymyxin B sulfate was purchased from Molekula GmbH (Munich, Germany) and sodium polyphosphate (Graham’s salt) was purchased from Merck KGaA (Vienna, Austria). Alkaline phosphatase from bovine intestinal mucosa (IAP, 7165 units/mg protein), ammonium molybdate tetrahydrate (81–83%), glucose-d-(+) ≥ 99.5% anhydrous, hydrochloric acid, sodium hydroxide, minimum essential medium (MEM) eagle, malachite green (MLG), oxalate salt 90%, potassium phosphate monobasic (KH_2_PO_4_) ≥ 99.5%, phosphatase inhibitor cocktail 2 (PIC), resazurin sodium salt, Triton X-100, lipase from porcine pancreas and fluorescein isothiocyanate isomer 1 (FITC) 90% were purchased from Sigma-Aldrich (Vienna, Austria). 4-(2-Hydroxyethyl)-1-piperazineethanesulfonic acid (HEPES) ≥ 99.5% was obtained from ROTH GmbH (Karlsruhe, Germany). d-(+)-Trehalose dihydrate >98.0% was purchased from TCI Chemicals (Eschborn, Germany). 2,4,6-Trinitrobenzenesulfonic acid (TNBS) was purchased from Chemos, Germany.

### 2.2 Methods

#### 2.2.1 FITC-Labeling of PMB

PMB was FITC-labeled via reaction between the amino groups of PMB and isothiocyanate group of FITC according to a previously described procedure with minor modifications.^13^ In brief, 40 mg (33.25 μmol) of PMB were dissolved in 10 mL of 100 mM HEPES buffer pH 7.4. In parallel, 17.2 mg of FITC (44.17 μmol) was dissolved in 1 mL of DMSO, added to the PMB solution, and constantly stirred at 300 rpm for 24 h under protection from light. The mixture was transferred into a dialysis membrane (Spectrum Spectra/Por, 1 kDa MWCO, Repligen, Germany) and dialyzed against DMSO for 24 h, followed by dialysis against water for 48 h. The final FITC-labeled PMB was lyophilized (Christ Gamma 1–16 LSC) and utilized to prepare fluorescently labeled PMB–PP NPs according to the method described below.

#### 2.2.2 Characterization of FITC-Labeled PMB

In order to determine the FITC-labeling efficiency of PMB, the degree of substitution (DS) of amino groups of PMB with FITC was evaluated using a TNBS assay based on a previously established method.^14^ 500 μL of DMSO was added to 0.5 mg of unmodified PMB and FITC-PMB. Samples were then diluted in 100 HEPES buffer pH 10 in the range of 0.015–0.125 mg/mL. Afterward, 100 μL of TNBS reagent (0.1% m/v TNBS in 8% (m/v) NaHCO_3_) was added to 100 μL of each previously diluted sample of PMB or FITC-PMB. The samples were covered by aluminum foil and incubated at 37 °C in an incubator for 2 h. Thereafter, the absorbance was measured at 420 nm. Free amine content was calculated by dividing the linear regression slope of each FITC-PMB with the slope of unmodified PMB. Degree of substitution (DS) was calculated as follows:
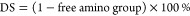


#### 2.2.3 Preparation of PMB–PP NPs

PMB–PP NPs were prepared via ionic gelation. PMB or FITC-labeled PMB was dissolved in 0.01 M HCl and pH was adjusted to 3 with 0.01 M NaOH. Demineralized water was added to obtain a final concentration of 1 mg/mL. PP was dissolved in demineralized water in a concentration of 1 mg/mL. The solutions were filtered using 0.2 μm cellulose acetate filters (Sartorius AG, Gottingen, Germany) before further use. One mL of PMB solution (1 mg/mL) was added dropwise into 2 mL (1 mg/mL) of PP solution under constant stirring at 800 rpm, followed by incubation at room temperature for 30 min. To prevent the formation of aggregates, 10 μL of 2% (m/v) trehalose was added to the suspension, that was centrifuged at 5500 g for 5 min with a MiniSpin Centrifuge (Eppendorf, Hamburg, Germany) in order to purify the obtained PMB–PP NPs. The supernatant was removed and NPs were resuspended in 1 mL of 100 mM HEPES buffer pH 7.4.

#### 2.2.4 Characterization of NPs

Particle size, PDI, and zeta potential of 0.01% (m/v) PMB–PP NPs in 100 mM HEPES buffer pH 7.4 were determined using a Zetasizer Nano ZS (Malvern Instrument, U.K.). Triplicated evaluations were carried out at 37 °C with a detection angle of 173°.

#### 2.2.5 Cytotoxicty Studies

Cytotoxicity studies were performed via resazurin assay on Caco-2 cells based on a previously described method.^15,16^ Prior to the experiment, cells were washed twice with 500 μL of glucose-HEPES buffer. % (m/v) to 0.01% (m/v). Thereafter, 500 μL of PMB–PP NPs were added to the wells and incubated at 37 °C for 2, 4, and 24 h. Glucose-HEPES buffer and 2% (m/v) Triton X-100 solution served as negative and positive control, respectively. At predetermined time points, cells were washed twice with 500 μL of a prewarmed glucose-HEPES buffer. Afterward, 250 μL of 2.2 mM resazurin solution were added to each well and incubated at 37 °C under light protection for 3 h. Thereafter, aliquots of 100 μL from each well were transferred to a 96-well black plate and fluorescence intensity was measured at an excitation wavelength of 540 nm and an emission wavelength of 590 nm using a microplate reader (Tecan Infinite M200; Grödig, Austria). Cell viability was calculated by the following equation:
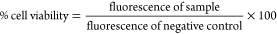


#### 2.2.6 Degradation Study with Lipase

Enzymatic degradation of free PMB and PMB being incorporated into NPs was evaluated with lipase. Lipase solution was prepared by dissolving 0.5 g of the enzyme in 10 mL of digestion medium (2 mM Tris buffer with 5 mM CaCl_2_ and 150 mM NaCl). After homogenization for 10 min, the enzyme mixture was centrifuged at 12,000 rpm (Sigma 3-18KS, Austria) for 15 min at 4 °C.^17^ Thereafter, 2 mL of the supernatant liquid was added to 4 mL of 0.01% (m/v) PMB–PP NPs suspension and incubated at 37 °C under constant shaking using a ThermoMixer (Eppendorf, Hamburg, Germany). Aliquots of 100 μL were withdrawn at predetermined time points (0, 1, 2, 4, 6, 12, and 24 h) and transferred to a 96-well microplate (μClear 96-well plates, Greiner bio-one, Austria). PMB–PP NPs and PMB suspension without lipase served as negative control and were incubated under the same conditions. Absorbance was measured at a wavelength of 210 nm using a microplate reader (Tecan Infinite M200; Grödig, Austria). The same settings were applied for PMB. The PMB concentration was determined using a calibration curve with increasing concentrations of PMB ranging from 0.78 to 50 μM.

Degradation was evaluated by quantifying the % remaining amount of PMB by the following equation:
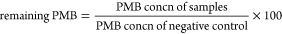


#### 2.2.7 Mucus Permeation Studies

Permeation behavior of PMB–PP NPs through the mucus gel layer was evaluated by the transwell insert method according to a previously described method with modifications.^18,19^ Mucus from porcine intestines was utilized after mucus purification process.^16^ In brief, transwell inserts with 33.6 mm^2^ surface area and 3 μm pore size (Greiner Bio-one, Austria) were placed on a 24-well plate and covered with 60 mg of porcine mucus. The acceptor chamber of each well was filled with 750 μL of 100 mM HEPES buffer pH 7.4. The donor chambers were filled with 250 μL of FITC labeled 0.05% (m/v) PMB–PP NPs or PMB suspensions in 100 mM HEPES buffer pH 7.4 or buffer only. Afterward, samples were incubated at 37 °C under continuous shaking at 300 rpm (Vibramax 100, Heidolph Instruments, Schwabach, Germany) under light protection. Aliquots of 100 μL were withdrawn from each well and replaced with the same volume of prewarmed buffer solution (100 mM HEPES buffer pH 7.4) at predetermined time points (0, 1, 2, 3, and 4 h). Fluorescence of the aliquots was measured in a 96-well plate (λ_ex_ = 488 nm and λ_em_ = 525 nm) using a microplate reader (Tecan Infinite M200; Grödig, Austria). The transwell inserts without mucus gel layer and without PMB–PP NPs or PMB suspensions served as positive and negative controls, respectively. The amount of PMB–PP NPs that permeated the mucus gel layer was calculated based on the values of positive and negative controls, including cumulative corrections.

#### 2.2.8 Enzymatic Phosphate Cleavage by Isolated IAP

Phosphate cleavage from PMB–PP NPs was evaluated by MLG assay using isolated IAP-. In brief, 12 μL of IAP solution (1 U/μL) was added to 6 mL of 0.01% (w/v) PMB–PP NPs in 100 mM HEPES buffer pH 7.5 and incubated at 37 °C under constant shaking at 300 rpm using a Thermomixer C (Eppendorf, Hamburg, Germany). PMB–PP NPs suspensions without IAP were incubated under equal conditions as control. Aliquots of 50 μL were transferred to a 96-well plate at predetermined time points (0, 1, 2, 3, and 4 h). In order to cease IAP activity after sampling, 5 μL of 3.6 M H_2_SO_4_ was added to each withdrawn aliquot. Phosphate release was evaluated by MLG assay as described below.

#### 2.2.9 Malachite Green Assay

In order to quantify the released monophosphates from PMB–PP NPs, MLG assay was performed. This assay quantifies just monophosphates without interfering with polyphosphate.^16^ Accordingly, colorimetric reagent solution was prepared by dropwise addition of 4.5 mL of 8% (m/v) ammonium molybdate solution to 8.5 mL of 0.15% (m/v) malachite green solution in 3.6 M of H_2_SO_4_ under high speed stirring and 340 μL of Triton X-100 in a concentration of 11% (m/v) was added to maintain the color. Subsequently, 100 μL of MLG reagent solutions were added to 50 μL of samples in 96-well plates and absorbance was measured at 630 nm using a microplate reader (Tecan Infinite M200; Grödig, Austria). The amount of released monophosphate was determined using a calibration curve with increasing concentrations of KH_2_PO_4_ ranging from 0.78 to 50 μM_._

#### 2.2.10 Enzymatic Phosphate Cleavage on Caco-2 Cells

Caco-2 cells were purchased from the European collection of cell cultures (ECACC, health protection agency, U.K.). Cells were cultured in 24-well plates at a density of 25000 cells/well with minimum essential medium (MEM) supplemented with 10% fetal bovine serum (FBS) and 1% penicillin-streptomycin at 37 °C and 5% CO_2_ atmosphere (95% relative humidity). The culture medium was changed on alternate days until a cell monolayer was observed. Prior to the experiment, cells were washed twice with 500 μL of glucose-HEPES buffer (268 mM glucose and 25 mM HEPES) pH 7.4. Afterward, cells were incubated with 500 μL of 0.01% (m/v) of PMB–PP NP suspensions in glucose-HEPES buffer pH 7.4. Aliquots of 50 μL were transferred to a 96-well plate at set time intervals (0, 1, 2, 3, and 4 h). In parallel, the experiment was performed in the presence of phosphatase inhibitor cocktail (PIC) (1% v/v) in glucose-HEPES buffer under the same conditions serving as control. Released phosphate was evaluated via MLG assay as described above.

#### 2.2.11 Drug Release Studies

To evaluate the release of PMB as a model peptide antibiotic from NPs, TNBS assay was utilized upon enzymatic cleavage of phosphate groups by IAP based on a previously described method with minor modifications.^20^ Accordingly, 6 mL of 0.01% (m/v) PMB–PP NPs in 100 mM HEPES buffer pH 7.4 was incubated with 12 μL of isolated IAP (1 U/μL). At predetermined time points, aliquots of 70 μL were withdrawn and 70 μL of TNBS reagent were added under constant shaking at 300 rpm for 90 min at 37 °C using a ThermoMixer (Eppendorf, Hamburg, Germany). Thereafter, 50 μL of aliquots were transferred into 96 microtiter plates at predetermined time points (0, 1, 2, 3, and 4 h) and absorbance was measured at 420 nm using a microplate reader (Tecan Infinite M200; Grödig, Austria). The amount of released PMB was evaluated using a calibration curve generated with increasing concentrations of PMB ranging from 0.78 to 50 μM. As control PMB–PP suspensions were incubated without the addition of isolated IAP and treated under the same conditions.

#### 2.2.12 Statistical Data Analysis

The statistical analysis of data was carried out by Graph Pad Prism 5.01 software. Mean ± standard deviation (SD) of the results were based on at least three experiments. Unpaired student’s *t* test was employed to analyze the differences between two independent groups. The level of significance was set as follows: *p < 0.05, **p < 0.01, and ***p < 0.005.

## 3 Results and Discussion

### 3.1 Preparation and Characterization of PMB–PP NPs

**Figure 1:**
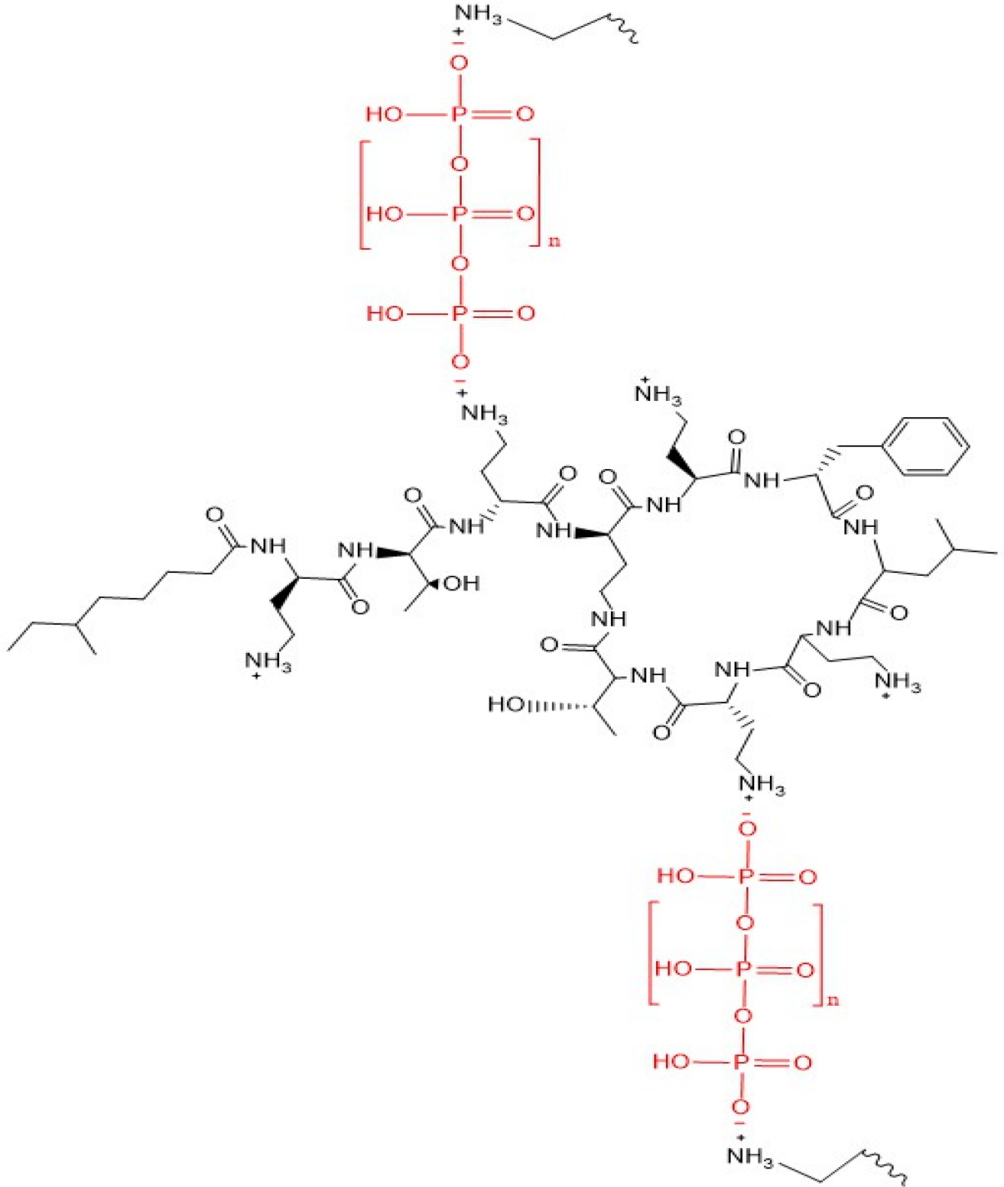
Schematic illustration of PMB–PP NPs prepared by ionic gelation.

PMB as a polycationic polypeptide poses challenges from the delivery point of view as it interacts with anionic endogenous substructures such as mucus glycoproteins on the way to its target. This so-called “polycation dilemma”^21^ causes low bioavailability and limits its therapeutic efficacy.^22−24^ In particular, mucosal delivery of PMB is therefore challenging. PMB–PP NPs were formed via ionic gelation as displayed in Figure 1.

As displayed in Table 1, NPs were obtained using different mass ratios between positively charged PMB to negatively charged PP to optimize the ionic cross-linking. Owing to the highly charged nature of polyphosphates at physiological pH^25^ they have been used to form polymeric NPs with oppositely charged polycations through ionic cross-linking resulting in particles with a small size and low polydispersity.^16^ Moreover, PP being regarded as safe auxiliary agents have been used in food industry as well^26^ and their mucus-permeating properties have already been reported.^27,28^

**Table 1:** Characterization of PMB–PP NPs Obtained by In Situ Gelation at Indicated Ratios

formulation code	PMB–PP ratio (w/w)	size (nm)	PDI	zeta potential (mV)
F1	1:1	202.53 ± 1.83	0.37	–19.17 ± 6.56
F2	1:2	197.13 ± 14.13	0.36	–11.09 ± 3.41
F3	1:3	727.95 ± 28.07	0.49	–14.90 ± 2.03

The ionic strength of the polycation plays also an important role in the formation of such particles. Since PMB exhibits p*K*_a_ values of 9.07, 9.54, 9.84, 10.02, and 10.24 and an isoelectric point of 10.74, it is a highly positively charged peptide at pH 7.4.^14^ PMB–PP NPs could therefore be formed.

As shown in Table 1, F2 exhibited the lowest particle size and PDI, and further addition of PP (m/m) did not result in a decrease in particle size and PDI (F3). In contrast, even larger particles of higher polydispersity were formed. Hence, F2 was chosen for further studies and referred as PMB–PP NPs, since it displays the smallest particle size and PDI along with a less negative charge being preferred for effective mucus permeation. Furthermore, F2 displayed a less negative initial zeta potential and a more pronounced charge reversal in orientating experiments. Moreover, particles exhibiting a moderate negative zeta potential with a high concentration of positive and negative charges on their surface showed high mucus diffusivity as they mimic the surface of viruses which are known to display high mucus permeating properties.^29^

### 3.2 Cytotoxicity of PMB–PP NPs

**Figure 2:**
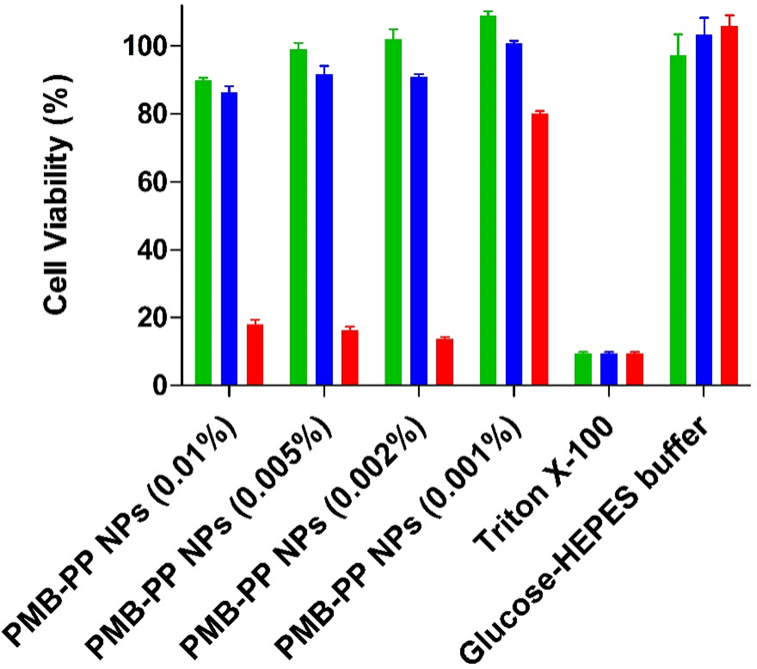
Viability of Caco-2 cells determined by resazurin assay after incubation with indicated concentrations of PMB–PP NPs. Green bars depict viability after incubation for 2 h, blue bars depict viability after incubation for 4 h and red bars depict viability after incubation for 24 h. Data are indicated as means ± SD (*n* = 3).

The cytotoxic potential of PMB–PP NPs was investigated on Caco-2 cells using a resazurin assay.^27,30^ The toxicity profile of PMB–PP NPs was investigated in concentrations ranging from 0.001% to 0.01% m/v, as shown in Figure 2.

As illustrated in Figure 2, toxicity was lowest at a concentration of 0.001% (w/v) after 2 h of incubation. However, cell viability declined to 80.14% ± 0.71 when cells were incubated with 0.001% (w/v) PMB–PP NPs for 24 h. Generally, all formulations were nontoxic within 2 h of incubation displaying a cell viability ≥85%.^31^ Cell viability decreased to ≤85% within 24 h of incubation with increasing concentrations of all formulations. The decrease in cell viability by prolonging incubation time can be explained by cleavage of PP by IAP over time releasing PMB that enhances cytotoxicity by interacting with negatively charged cell membranes.^32^ Caco-2 cells displaying a viability of >80% when incubated with lipid formulations were reported to well-tolerate these formulations.^33^ Moreover, according to ISO 10993–5, when *in vitro* toxicity of compounds against mammalian cells is investigated, percentages of cell viability above 80%, within 80%–60%, within 60%–40% and below 40% are considered as noncytotoxic,weak, moderate and strong cytotoxic, respectively.^31^ Polymyxins demonstrate their bactericidal effects mostly against Gram-negative bacteria.^12^ In a recent study, authors evaluated the MICs (minimum inhibitory concentrations) of PMB against 50 multidrug-resistant Gram-negative bacterial strains, and MICs were reported to be ≥4 μg/mL for 44 of the strains and were 2 μg/mL for the other 6 strains.^34^ Within this study, we utilized NP concentrations between 0.001% (m/v) to 0.01% (m/v) for PMB–PP NPs which refer to ∼3.33–33.3 μg/mL PMB that would be effective against the mentioned Gram-negative bacterial strains without causing toxic effects for mammalian cells for upto a 4h application time.

These results were also in a good agreement with a previous study carried out by Jalil et al. showing decreased cell viability (≤80%) of self-emulsifying drug delivery systems containing PMB on Caco-2 cell monolayer within 24 h.^35^

### 3.3 Protective Effect of PMB–PP NPs toward Enzymatic Degradation

**Figure 3:**
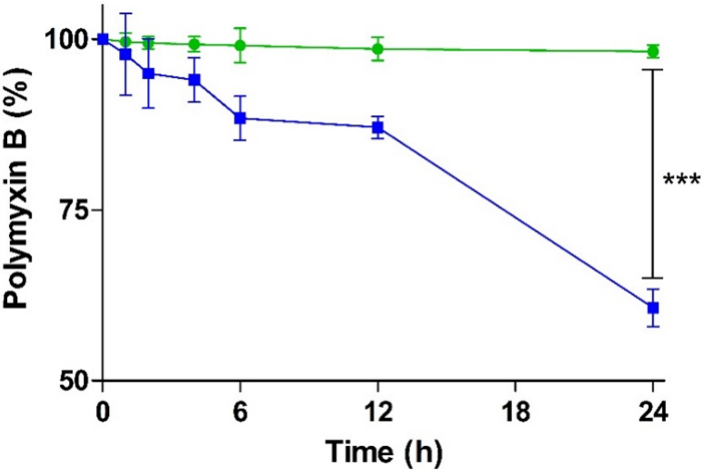
Degradation of PMB–PP NPS (green circles) and PMB (blue squares) by lipase incubated in HEPES buffer pH 7.4 at 37 °C. Data are indicated as means ± SD (*n* = 3).

In contrast to most other polypeptide antibiotics that are mainly degraded by peptidase and proteases, PMB is predominantly degraded by lipase.^7^ The degradation of PMB in the form of PMB–PP NPs and free PMB is shown in Figure 3.

As illustrated in Figure 3, free PMB was degraded to a high extent (39.32%) after lipase exposure. Since in the small intestine bile salts and colipase will accelerate this enzymatic reaction, degradation of PMB will be *in vivo* likely even more pronounced. In contrast, PMB–PP NPs showed effective protection retaining approximately 97% of intact PMB under simulated physiological conditions for 24 h. These findings are in good agreement with previous reports mentioning that PMB is rapidly degraded at pH 7.4 by lipase under physiological conditions.^7,36^ Likely because of their electrostatically cross-linked stable network, PMB–PP NPs provided a protection for PMB against enzymatic degradation by lipase.^37^ Furthermore, it has been demonstrated that usage of polymers can improve the bioavailability of peptides by providing protection against enzymatic degradation.^38,39^

### 3.4 Mucus Permeation Behavior of PMB–PP NPs

**Figure 4:**
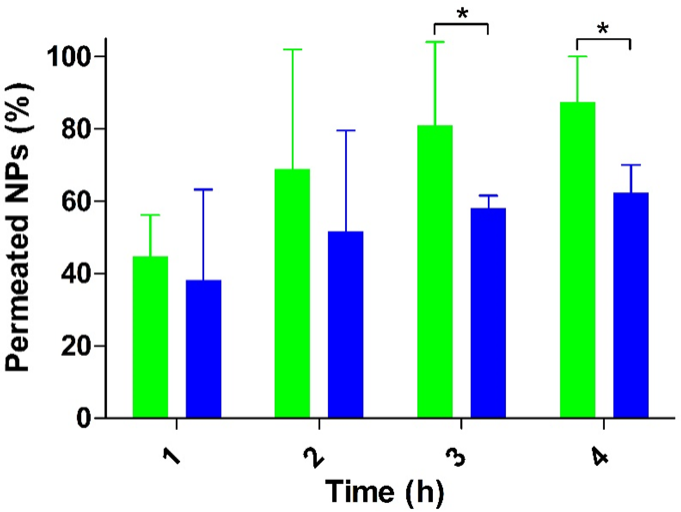
Mucus permeation studies of PMB–PP NPs using the transwell insert method with porcine intestinal mucus. Green bars indicate PMB–PP NPs and blue bars indicate PMB. The transwell inserts without a mucus gel layer served as positive control (100% control value). Data indicated as means ± SD (*n* = 3). (*p < 0.05).

Mucus permeation studies were performed using a transwell insert method employing porcine intestinal mucus as barrier. The transwell insert method is an established setup for mucus diffusion studies of NPs to predict their *in vivo* permeation behavior.^18,40,41^ Results are shown in Figure 4.

As illustrated in Figure 4, PMB–PP NPs demonstrated a 1.4-fold higher mucus permeation than PMB within 4 h. According to these results, the negative surface charge of NPs facilitated the permeation of PMB across the mucus gel layer. A recent study reported that PP contributed to mucus permeation of NPs due to increased electrostatic repulsion forces against the negatively charged sialic and sulfonic acid moieties of the mucus gel layer.^16,42^ PMB interaction with target bacterial cells occurs mainly via electrostatic interactions.^12^ Since the number of amino groups on PMB is lowered by the covalent attachment of FITC, the cationic character of the drug will decrease. In order to minimize this effect, on average just 7.2% of amino groups on PMB were modified. Moreover, similar to PMB–PP NPs, virus-mimicking nanocarriers displaying a high concentration of positive and negative charges on their surfaces have been shown to diffuse the mucus gel layer as fast as in saline and most promising results were obtained when relatively more negative virus-mimicking NPs were utilized.^29^

### 3.5 Phosphate Release from PMB–PP NPs

#### 3.5.1 Enzymatic Phosphate Cleavage by Isolated IAP

**Figure 5:**
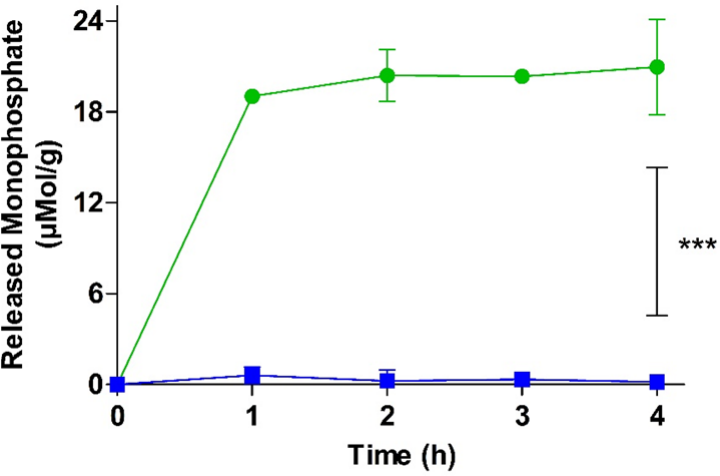
Monophosphate release from PMB–PP NPs in the presence (green circles) and absence (blue squares) of isolated IAP (2 U/mL) at 37 °C. Data are indicated as means ± SD (*n* = 3). ***p ≤ 0.001.

As a membrane-bound catalytic enzyme, the ability of IAP to cleave phosphate esters of alcohols and phenols has been shown in numerous studies.^16,19,43^ Furthermore, it has been reported that polyphosphate can be cleaved by IAP.^44−46^ Polyphosphate cleavage from PMB–PP NPs was investigated using isolated IAP by determining phosphate release over time. The activity of IAP was determined in human small intestinal mucosa within 12 h after death with 796 units per 100 g of mucosa.^47^ Within our study we applied a 24-fold lower phosphatase activity (0.33 units/mL) taking a lower accessibility of this membrane bound enzyme under *in vivo* conditions into consideration. A time-dependent phosphate release from PMB–PP NPs was observed as shown in Figure 5.

A significantly increased phosphate release over a time period of 4 h was observed. A total amount of 20.96 μmol/g monophosphates was released from PMB–PP NPs within 1 h, followed by a plateau phase. In the absence of IAP, negligible amounts of monophosphate was released. These results showed that isolated IAP can even cleave polyphosphate on the surface NPs. This result is in agreement with previous studies showing that polyphosphates used as coating for nanoemulsions^48^ and nanostructured lipid carriers (NLCs) are also cleaved by IAP.^27,49,50^ Similarly, in a previous study, authors developed polyethylene imine (PEI)–PP NPs to overcome the mucus gel layer and to enable a charge conversion at the epithelial cell membrane showing monophosphate release from these particles in the presence of IAP.^16^

#### 3.5.2 Enzymatic Phosphate Cleavage by Caco-2 Cells

**Figure 6:**
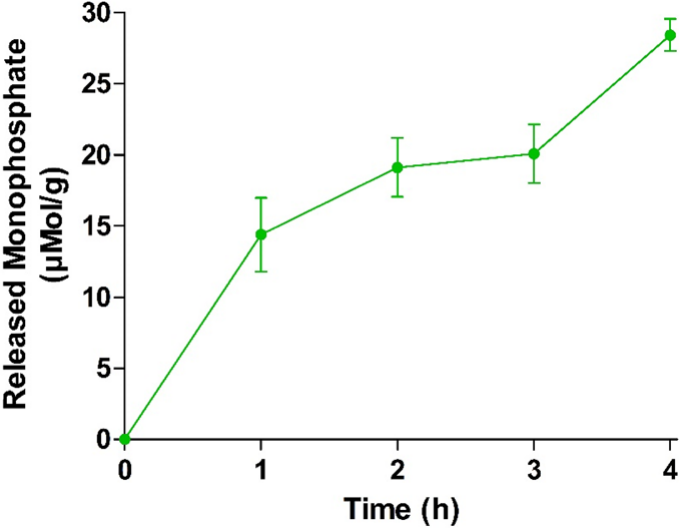
Monophosphate release from PMB–PP NPs diluted in 268 mM glucose and 25 mM HEPES buffer pH 7.4 at 37 °C, mediated by enzymatic cleavage with IAP expressed on the Caco-2 cell monolayer. Data are indicated as means ± SD (*n* = 3).

Phosphate release from nanocarriers upon incubation with Caco-2 cells was shown in numerous *in vitro* studies.^15,16,51^ Caco-2 cells bear IAP on their outer cell membrane cleaving phosphate groups.^4,15,19,52^ In order to evaluate monophosphate release from PMB–PP NPs via membrane bound IAP, Caco-2 cells were utilized. As illustrated in Figure 6, results showed that monophosphate release increased in a time-dependent manner.

Within 1 h, 14.4 ± 2.6 μmol of monophosphate were released from one gram (g) of PMB–PP NPs. After 4 h, 28.4 μmol of monophosphate were released per g of PMB–PP NPs. However, in case of control groups, no statistically significant amount of monophosphate was released throughout the entire experiment (data not shown). These results confirm that PMB–PP NPs reach membrane-bound IAP at the intestinal brush border membrane leading to phosphate cleavage.^27^ Results are in good agreement with a previous study carried out by Nazir et al. using chitosan and chondroitin sulfate to form nanoparticles that were additionally phosphorylated. Incubation of these NPs with Caco-2 cells resulted also in a time dependent release of monophosphate.^19^

#### 3.5.3 Enzyme-Mediated Zeta Potential Change

**Figure 7:**
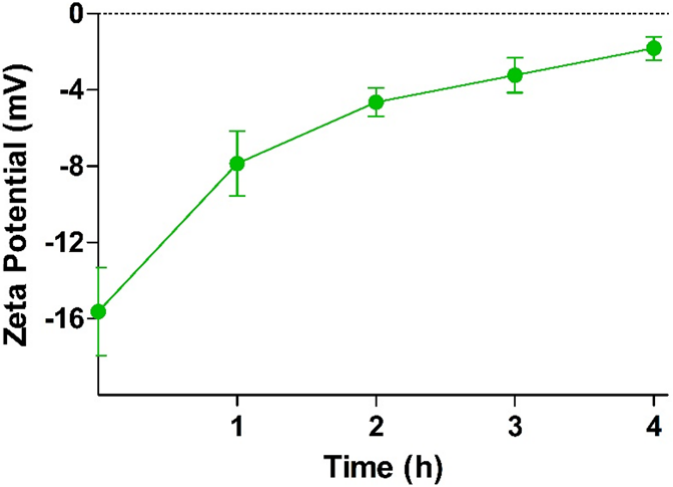
Time-dependent zeta potential change of PMB–PP NPs after enzymatic cleavage of phosphate groups using isolated IAP- (2 U/mL) at 37 °C. Data are indicated as means ± SD (*n* = 3).

Isolated IAP was utilized to cleave phosphate moieties from the surface of PMB–PP NPs. The change in zeta potential was monitored over 4 h. The results are shown in Figure 7.

The trend of monophosphate release is in accordance with the zeta potential change. In detail, a change in zeta potential from −15.6 mV to −1.9 mV was observed upon incubation of PMB–PP NPs with IAP. Likewise, changes in zeta potential as a function of time indicate a loss of anionic moieties from the surface of the particles. The results were in good agreement with previous findings of Suchaoin et al. showing a change in zeta potential of phosphorylated carboxymethyl cellulose–glucosamine 6-phosphate (CMC–G6P) polyethylene imine–polyarginine conjugate NPs caused by phosphate release in the presence of IAP.^53^ Accordingly, these current results provide evidence that IAP is capable of cleaving phosphate groups from PMB–PP NPs, thus demonstrating the applicability of this well-established concept to NPs formed in this study.

### 3.6 Drug Release from PMB–PP NPs

PMB release from PMB–PP NPs was evaluated upon cleavage of phosphate groups by IAP-stimuli revealing the primary amino groups of PMB that can be quantified by TNBS assay.

**Figure 8:**
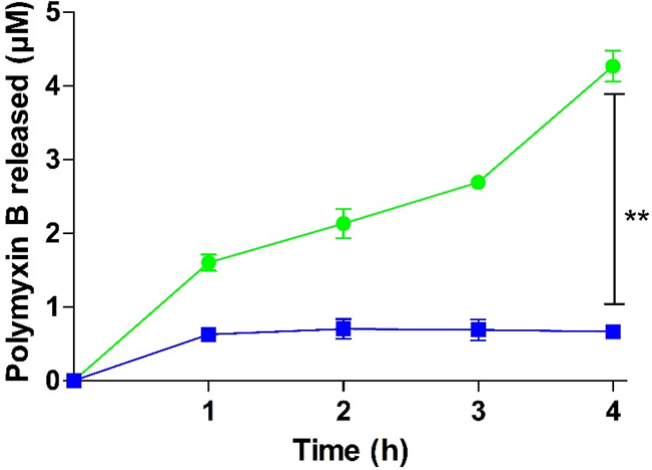
Release of PMB from PMB–PP NPs by enzymatic phosphate cleavage using isolated IAP (2 U/mL) at 37 °C (green circles) as well as under the same conditions but omitting isolated IAP (blue squares). Data are indicated as means ± SD (*n* = 3).

Results are illustrated in Figure 8.

During incubation with IAP, a significant release of PMB from PMB–PP NPs in a time-dependent manner was observed. In contrast, only a negligible amount of PMB was released in case of the control without IAP. Since PMB–PP NPs are formed via electrostatic interactions between cationic PMB and anionic PP, enzymatic degradation of PP to monophosphate causes the disintegration of the carrier system and release of PMB. Upon incubation with isolated as well as cell-derived IAP, monophosphates were released from PMB–PP NPs within 4 h, as shown in Figures 5 and 6, respectively. There is almost no release of monophosphates when enzyme activity was inhibited, indicating that cleavage of PP was IAP-dependent. A similar release pattern was observed for PMB (Figure 8), whereas without IAP, almost no PMB was released. Furthermore, upon incubation with IAP, the zeta potential of PMB–PP NPs changed from negative to almost neutral values, indicating that the enzyme was able to cleave the anionic PP on the surface of PMB–PP NPs, revealing the underlying cationic PMB. These results were in good agreement with previous findings of Leichner et al., demonstrating the release of β-galactosidase from chitosan–tripolyphosphate NPs after IAP stimuli.^4^ Furthermore, IAP triggered also a significant release of rhodamine 123 from chitosan–polyphosphate NPs under physiological conditions.^5^

## 4 Conclusions

Within this study, PMB–PP NPs were developed using PMB as cationic model polypeptide antibiotic through ionic gelation with PP to provide protection against enzymatic degradation (i), to overcome the mucus gel barrier (ii), and to provide a targeted drug release at the epithelium (iii). PMB–PP NPs exhibited a size around 200 nm and a negative surface charge. Degradation of PMB under physiological conditions was prevented by incorporation in these NPs. A concentration- and time-dependent cytoxicity was observed. NPs were able to cross the mucus gel layer as a result of their zwitterion surface, mimicking the surface charge characteristics of viruses. Furthermore, an IAP-dependent cleavage of PP from PMB–PP NPs was shown. In parallel, a change in zeta potential of PMB–PP NPs was observed, providing evidence for the release of phosphate groups from the NPs. IAP-dependent cleavage of PP simultaneously enabled the release of PMB from PMB–PP NPs. According to these results, the incorporation of cationic peptide antibiotics in biodegradable PP NPs holds promise to improve their efficacy to eradicate pathogenic microorganisms within the intestinal mucosa and to create the basis for their systemic delivery via the oral route.
